# Comparative transcriptome analysis provides insights into the molecular mechanisms of high-frequency hearing differences between the sexes of *Odorrana tormota*

**DOI:** 10.1186/s12864-022-08536-2

**Published:** 2022-04-12

**Authors:** Zhuo Chen, Yao Liu, Rui Liang, Chong Cui, Yanjun Zhu, Fang Zhang, Jie Zhang, Xiaohong Chen

**Affiliations:** 1 College of Life Sciences, Henan Normal University, Xinxiang, 453007 China; 2 The Observation and Research Field Station of Taihang Mountain Forest Ecosystems of Henan Province, Xinxiang, 453007 China; 3 College of Life Sciences, Anhui Normal University, Wuhu, 241000 China; 4 College of Fisheries, Henan Normal University, Xinxiang, 453007 China

**Keywords:** *Odorrana tormota*, High-frequency hearing, Adaptation, Sex dimorphism, Transcriptome

## Abstract

**Background:**

Acoustic communication is important for the survival and reproduction of anurans and masking background noise is a critical factor for their effective acoustic communication. Males of the concave-eared frog (*Odorrana tormota*) have evolved an ultrasonic communication capacity to avoid masking by the widespread background noise of local fast-flowing streams, whereas females exhibit no ultrasonic sensitivity. However, the molecular mechanisms underlying the high-frequency hearing differences between the sexes of *O. tormota* are still poorly understood.

**Results:**

In this study, we sequenced the brain transcriptomes of male and female *O. tormota*, and compared their differential gene expression. A total of 4,605 differentially expressed genes (DEGs) between the sexes of *O. tormota* were identified and eleven of them were related to auditory based on the annotation and enrichment analysis. Most of these DEGs in males showed a higher expression trend than females in both quantity and expression quantity. The highly expressed genes in males were relatively concentrated in neurogenesis, signal transduction, ion transport and energy metabolism, whereas the up-expressed genes in females were mainly related to the growth and development regulation of specific auditory cells.

**Conclusions:**

The transcriptome of male and female *O. tormota* has been sequenced and de novo assembled, which will provide gene reference for further genomic studies. In addition, this is the first research to reveal the molecular mechanisms of sex differences in ultrasonic hearing between the sexes of *O. tormota* and will provide new insights into the genetic basis of the auditory adaptation in amphibians during their transition from water to land.

**Supplementary Information:**

The online version contains supplementary material available at 10.1186/s12864-022-08536-2.

## Background

Acoustic communication is widespread and essential in many tetrapods (e.g., frogs, birds and mammals) [1, 2]. The auditory system is responsible for detecting and processing airborne sound signals, which is important for survival and reproduction of terrestrial vertebrates [3]. Generally, the auditory system includes the outer ear (the auricle and auditory canal), the middle ear (the tympanic membrane, malleus, incus and stapes), the inner ear (the cochlea and vestibular organs), the auditory nerve, the auditory cortex and other brain areas involved in sound processing [4]. The effective detection and transmission of sound signals via the auditory system facilitate the acoustic communication [5–7]. However, the ambient noise could affect the transmission efficiency of sound signals in many vocal vertebrates due to the overlapping sound waves (e.g., frequency and amplitude) with animal sound [8, 9]. To minimal masking background noise, some animals (e.g., whales, dolphins, bats and rodents) evolved ultrasonic communication [10]. Ultrasonic communication benefits these animals via enhanced signal-to-noise ratio, avoidance of eavesdropping by predators or prey, and increased energetic efficiency [11, 12].

Generally, most anuran amphibians (i.e., frogs and toads) produce repetitive, highly stereotyped calls containing frequency components between ~ 100 Hz and 5–8 kHz [13–16]. However, electrophysiological recordings and acoustic playback experiments showed that three frog species (i.e., *Odorrana tormota*, *Huia cavitympanum* and *Odorrana graminea*) can detect ultrasound (≧20 kHz) and use ultrasonic vocalizations for intraspecific communication [14, 17, 18]. Several hypotheses based on the comparison of habitats and morphologies were put forward to explain their ultrasonic adaptation [14, 15, 19]. All the three species inhabit rapid-flowing montane streams, and the evolution of ultrasound communication might be the adaptation to the intense, predominately low-frequency ambient noise from nearby streams and waterfalls [15]. The recessed tympana evolved in *O. tormota* and *H. cavitympanum* might aid their reception of ultrasound, whereas the tympanic membranes are not recessed in *O. graminea* [14]. Additionally, the closed state of the Eustachian tubes might facilitate the transmission of the ultrasonic from the middle ear to the inner ear in *O. tormota* [19].

The concave-eared torrent frogs (*O. tormota*), one of the endemic odorous frogs in China, is the first non-mammalian vertebrate demonstrated to communicate with ultrasonic frequencies (≧20 kHz) [17]. Calls of *O. tormota* contain an audible dominant frequency ranged from 5–7 kHz as in other frogs, but they also contain prominent ultrasound harmonics [17, 18]. Evidences suggested that only males of *O. tormota* have evolved the ultrasonic communication capacity and the upper frequency limit of the male could reach to 34 kHz, whereas females exhibit no ultrasonic sensitivity and the upper frequency limit of the female was at ~ 16 kHz [20]. Males of *O. tormota* can detect and use ultrasound to avoid masking by the wideband stream noise, communicate during male-male territorial interactions, accurately locate and attract females during the breeding season [17, 21]. The morphological difference that the tympanic membrane is recessed in males but not in females might explain the high-frequency hearing sexual polymorphism at some extent [22]. In addition, the thinner tympanic membrane and lower mass ossicles of males than females might contribute to the ultrasonic hearing in males [20]. However, the genetic changes underlying the high-frequency hearing differences between the sexes of *O. tormota* are still poorly documented.

Evidences indicated that the superior olivary nucleus (SON) in the hindbrain and principal nucleus of the torus semicircularis (Ptor) in *O. tormota* were considered as the high-frequency sensitive domains based on the expression analysis of *egr-1* via ultrasound-only call treatment [23]. Previous studies also suggested that different expression patterns of the key genes involved in the reception and transmission of auditory signals in the brain can influence the high-frequency hearing sensitivity [24, 25]. For example, the higher expressions of *TMC1* and *Otof* genes in the brain are essential for high-frequency hearing in echolocating bats and toothed whales [24, 25]. In addition, recent high-throughput RNA-Sequencing (RNA-Seq) technologies provided us a large-scale platform to address the molecular mechanisms underlying the adaptive evolution of particular phenotypes in non-model organisms [26–28]. For example, Sun et al. (2020) found that the expression level of *FBXL15* gene played an important role in changing the call frequency of horseshoe bats based on comparative transcriptome analysis [27].

In this study, we sequenced and de novo assembled the brain transcriptome of male and female *O. tormota* using next-generation sequencing technology. Our objectives were to: 1) compare the differential gene expression profiles between sexes of *O. tormota*; 2) analyze the biological information regarding the transcriptional profiles of the brain; 3) identify the candidate genes involved in the high-frequency hearing difference between sexes of *O. tormota*. The assembled transcriptome sequences will expand the genetic information for functional genomic studies of *O. tormota*. This is the first research to reveal the molecular mechanisms of sex differences in ultrasonic hearing between the sexes of *O. tormota* and will provide new insights into the genetic basis of the auditory adaptation in amphibians during their transition from water to land.

## Results

### Transcriptome sequencing and de novo assembly of *O. tormota*

To exclude the individual difference, we randomly mixed different individuals of the same sex. Two male (named M1 and M2) and two female (named F1 and F2) groups of *O. tormota* were randomly divided and used for de novo transcriptome sequencing. Illumina sequencing of all samples produced over 257 million paired-end raw reads, of which 64,310,120 reads for F1, 64,333,040 reads for F2, 64,548,500 reads for M1 and 64,382,860 reads for M2. After the removal of low-quality reads, poly-N-containing reads and adapters, 99.99% of the raw reads were generated as clean reads for each sample, suggesting the high quality of the sequencing data. The Q30 value of the four samples varied from 96.03% to 96.68% and the overall G/C content was 44.00% for each sample. The statistics of sequencing are summarized in Table 1. All raw sequence reads data have been deposited in the NCBI Gene Expression Omnibus (GEO) database under the accession number GSM5691958, GSM5691959, GSM5691960, and GSM5691961.

**Table 1:** Statistics of the sequencing for *Odorrana tormota* transcriptome

Sample	Clean reads	Valid ratio (base)	Q30 (%)	GC content (%)
F1	64,310,120	99.99%	96.68%	44%
F2	64,333,040	99.99%	96.28%	44%
M1	64,548,500	99.99%	96.26%	44%
M2	64,382,860	99.99%	96.03%	44%

Due to the lack of reference genome of *O. tormota*, we de novo assembled the transcriptome and used it as a reference for subsequent analysis. The high-quality clean reads for all the samples mentioned above were used for de novo transcriptome assembly of *O. tormota*. Finally, a set of 197,685 unigenes with an average length of 797.76 bp and N50 (sequence length of the shortest transcript at 50% of the total genome length) value of 1,006 bp was obtained (Additional file 2: Table S2). The longest unigene was 26,449 bp and the detailed length distribution of all unigenes is shown in Additional file 3: Fig. S1. Among the total number of clean reads from the four samples in this study, 83.31% to 84.21% were successfully mapped against the assembled unigenes. In addition, the percentage of the unique mapping reads of the clean reads to the assembled unigenes ranged from 68.48% to 70.20% (Additional file 4: Table S3).

### Functional annotation

The unigene functional annotations were conducted with a 10^–5^ e-value cut-off value based on the alignment to the five public databases, i.e., non-redundant protein sequence database (NR), the manually annotated and reviewed protein sequence database (Swiss-Prot), the eukaryotic Ortholog Groups (KOG), The Kyoto Encyclopedia of Genes and Genomes (KEGG) and Gene Ontology (GO). Approximately 26.22% (51,832 of 197,685) unigenes were significantly matched with one or more of the five databases (Table 2). Among them, a total of 49,222 unigenes (24.90%) were annotated against the NR database. The species distribution of NR BLAST matches is shown in Additional file 5: Fig S2, and the top three matched species were *Xenopus tropicalis* (42.34%), *Gallus gallus* (9.80%) and *X. laevis* (7.48%).

**Table 2:** Summary of the unigenes annotated in different databases

Database	Annotation number	Annotation ratio
NR	49,222	24.90%
SWISSPORT	41,085	20.78%
KOG	29,476	14.91%
KEGG	14,938	7.56%
GO	37,239	18.84%

**Fig. 1:**
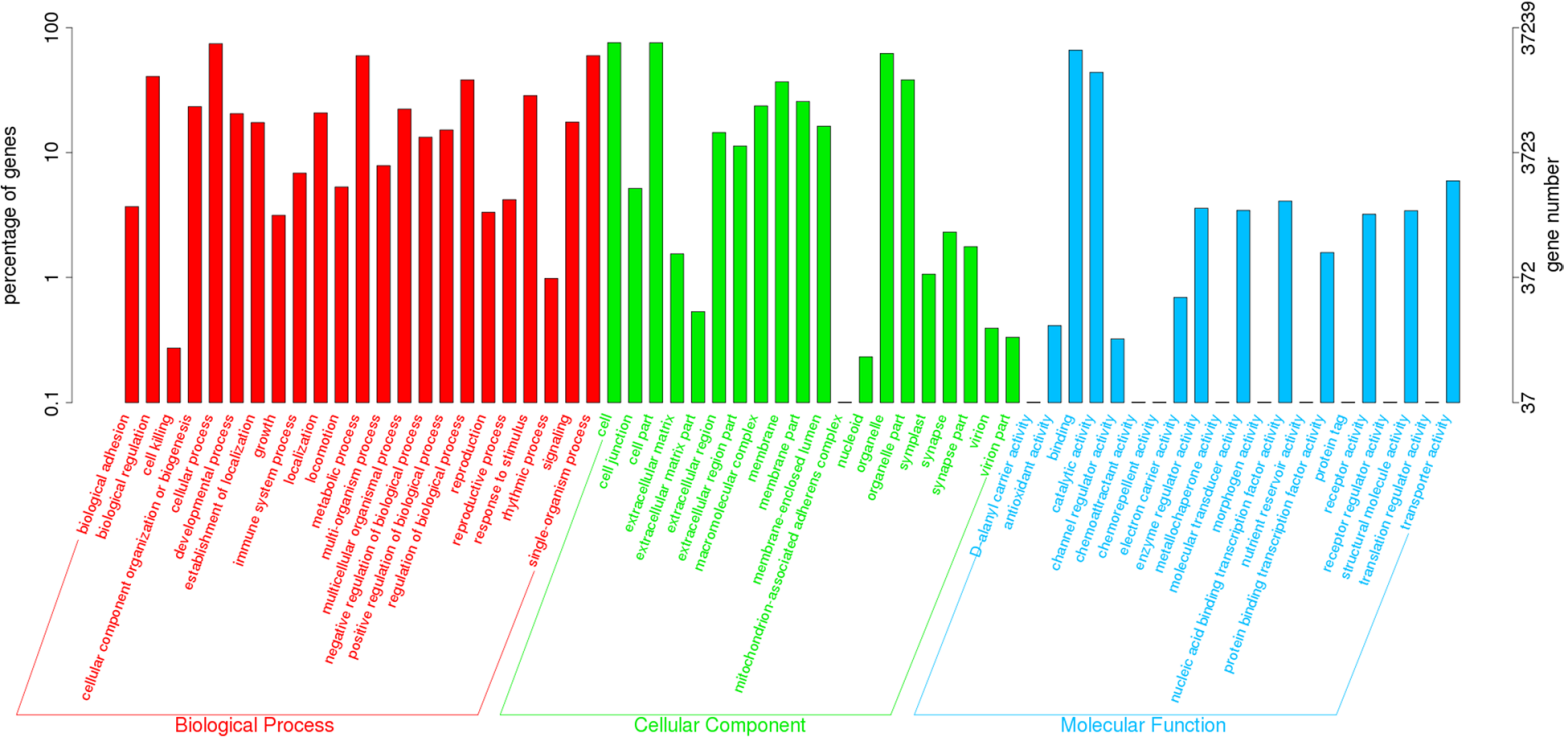
Gene Ontology (GO) classification of the assembled unigenes. Three main GO categories were shown in different colors (Red: Biological Process, Green: Cellular Component, Blue: Molecular Function). The GO terms name were shown at the horizontal axis, the gene number and percentage of unigenes were given at the vertical axis

The KOG annotation analysis showed that 14.91% (29,476 of 197,685) annotated unigenes was classified into 25 KOG categories. Among them, the largest group was “General function prediction”, followed by “Signal transduction mechanisms” and “Posttranslational modification, protein turnover, chaperones” (Additional file 6: Fig. S3). Additionally, 18.84% (37,239 of 197,685) unigenes were categorized into 64 subcategories of GO terms under three major categories: Biological Process (BP), Cellular Component (CC), and Molecular Function (MF). The predominant group in each of BP, CC and MF was cellular process (27,666 unigenes, 74.29%), cell (28,203 unigenes, 75.73%), and binding (24,561 unigenes, 65.95%), respectively (Fig. 1).

**Fig. 2:**
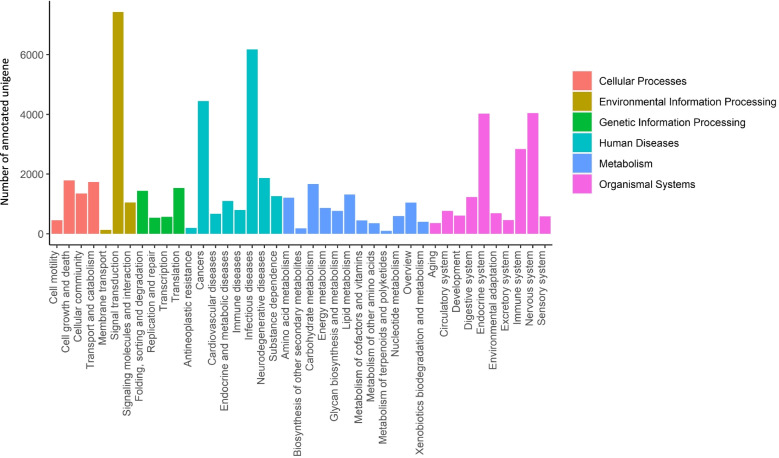
Histogram of KEGG pathway annotation of the unigenes in *O. tormota*. The six main KEGG classifications were shown in different colors as shown at the right side. The x-axis and y-axis represent the annotated pathway and the number of annotated unigenes, respectively

To further understand the biological functions and interactions of the transcripts, a total of 14,938 unigenes were mapped to 354 KEGG pathways and assigned to six major categories: Cellular Processes, Environmental Information Processing, Genetic Information Processing, Human Diseases, Metabolism and Organismal Systems. The most abundant category was “signal transduction”, followed by “infectious diseases”, “cancers”, “endocrine system”, and “nervous system” (Fig. 2). These annotation and predicted pathways will aid the understanding of the gene function in *O. tormota*.

### Identification and analysis of differentially expressed genes (DEGs)

To identify DEGs between different groups, the fragments of exon per kb per million fragments (FPKM) algorithm was used to compare relative transcript abundance in each unigene. The hierarchical clustering analysis revealed that the DEGs in the same gender have higher similarity and formed a sister-group relationship (Additional file 7: Fig S4), although 4,846 and 5,314 DEGs were identified between F1 and F2, M1 and M2 groups, respectively (Additional file 8: Table S4). We further explored the DEGs between male and female groups, and a total of 4,605 significantly DEGs were identified. Among these DEGs, 1,140 genes showed significantly higher expression levels in females than those in males, whereas 3,465 genes showed significantly lower expression levels in females than those in males (Additional file 9: Table S5).

**Fig. 3:**
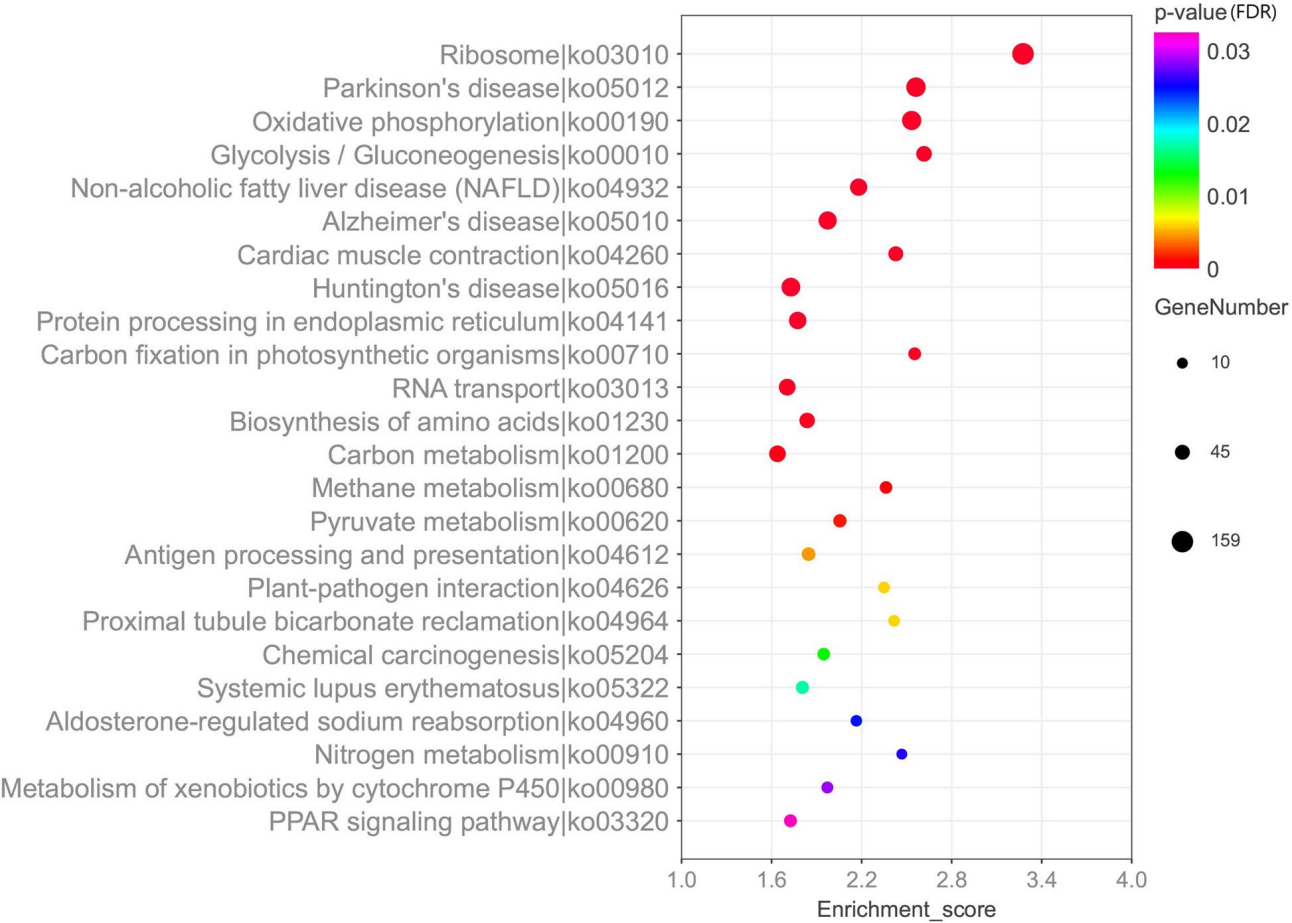
An overview of the KEGG pathways significantly enriched in DEGs. The horizontal axis indicates the enrichment scores of DEGs, and the specific pathways are plotted along the vertical axis. The color of each dot represents the corrected *P*-value for the corresponding pathway, and the dot size indicates the number of the DEGs associated with each corresponding pathway

Among the 4605 DEGs between sexes, 2,600 DEGs were significantly enriched in 1170 GO terms and most of those DEGs were assigned to two major categories: “Biological Process” and “Molecular Function”. Among the 2600 enriched DEGs, 2017 DEGs were up-regulated in the male group. The KEGG pathway enrichment analysis of the DEGs between sexes indicated that 1288 DEGs were significantly mapped to 24 KEGG pathways (Fig. 3). The abundant enriched categories were “Metabolism” and “Organismal Systems”. Among the metabolic category, most of the unigenes were down-regulated in females and they were mainly related to “amino acid metabolism”, “Glycolysis/Gluconeogenesis” and “energy metabolism processes”. As for the “Organismal system” category, most of the DEGs were up-regulated in females and they were mainly mapped to the pathways of “crucial signaling pathways” and “maintenance of homeostasis” (Additional file 10: Table S6).

### Characterization and expression pattern of auditory-related genes between sexes of *O. tormota*

**Fig. 4:**
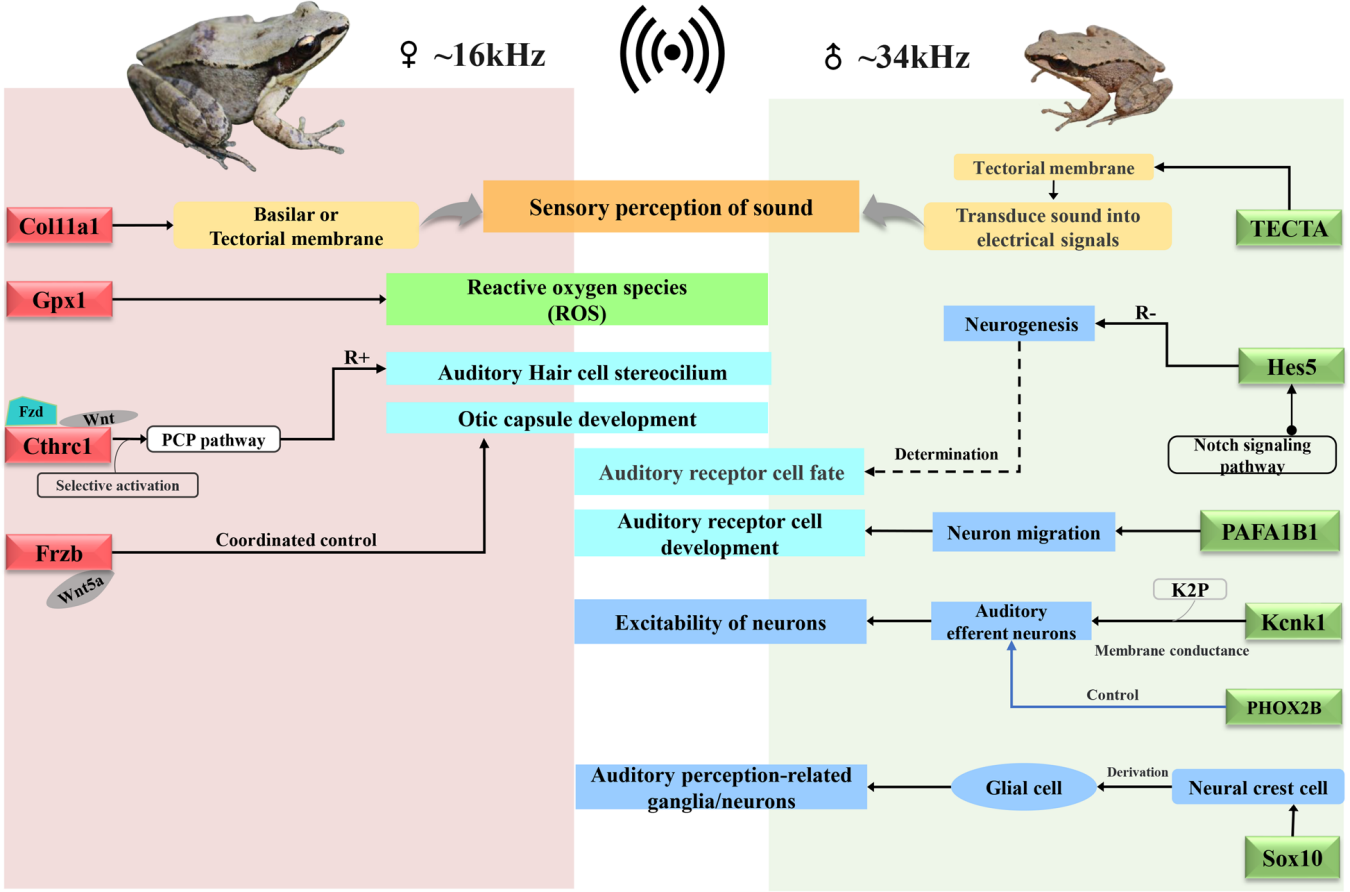
Schematic diagram for the functions of the auditory-related DEGs between sexes of *O. tormota*. The up-regulated DEGs in females were highlighted in red, and the up-regulated DEGs in males were highlighted in green. Note: R^+^ indicates positive regulation; R^−^ indicates negative regulation

To explore the genes controlling the high-frequency hearing difference between sexes of *O. tormota*, the auditory-related genes and their expression pattern were further analyzed. Among the 2,600 DEGs enriched in GO terms, eleven auditory-related genes (i.e., *GPX1*, *Cthrc1*, *Col11a1*, *Frzb*, *SOX10*, *TIMM13*, *HES5*, *PHXO2B*, *TECTA*, *PAFAH1B1* and *KCNK1*) were identified. These auditory-related genes were assigned to six GO terms (GO: 0071600, GO: 0090103, GO: 0048752, GO: 0048840, GO: 2000981, GO: 0042668) and they were involved in the auditory system morphogenesis, differentiation, sensory perception of sound, and mechanoelectrical transduction associated with hearing signal transduction (Table 3). Four of the eleven genes (i.e., *GPX1*, *Cthrc1*, *Col11a1* and *Frzb*) were significantly up-regulated in females, whereas the other seven genes (i.e., *SOX10*, *TIMM13*, *Hes5*, *PHXO2B*, *TECTA*, *PAFAH1B1* and *KCNK1*) were highly expressed in males (Additional file 11: Table S7). In addition, the expression level of the typical high-frequency sensitive hearing genes (e.g. *KCNQ4* and *Prestin*) revealed in previous studies exhibited no significant difference between sexes of *O. tormota*. The regulatory pathway of the auditory-related genes revealed in this study are shown in Fig. 4 [29, 30]. Interestingly, both the number of DEGs and the expression levels in males was higher than that in females, suggesting the existence of the expression differences of the auditory-related genes between sexes of *O. tormota.*

**Table 3:** The significant GO terms related to auditory of DEGs in *O. tormota*

GO ID	GO Term	Category	*P*-Value	List Hits	Gene ID
GO:0071600	otic vesicle morphogenesis	P	0.0139	1	comp228205_c2_seq3
GO:0090103	cochlea morphogenesis	P	0.0146	2	comp213035_c0_seq1 comp227468_c5_seq8
GO:0048752	semicircular canal morphogenesis	P	0.0423	1	comp228205_c2_seq3
GO:0048840	otolith development	P	0.0423	1	comp228205_c2_seq3
GO:2000981	negative regulation of inner ear receptor cell differentiation	P	0.0423	1	comp230058_c2_seq5
GO:0042668	auditory receptor cell fate determination	P	0.0423	1	comp230058_c2_seq5

Note: “P” represents “Biological Processing”, the “List Hits” are the differential unigene numbers in this GO term

### Validation of differential gene expression by quantitative real time PCR (qRT-PCR)

**Fig. 5:**
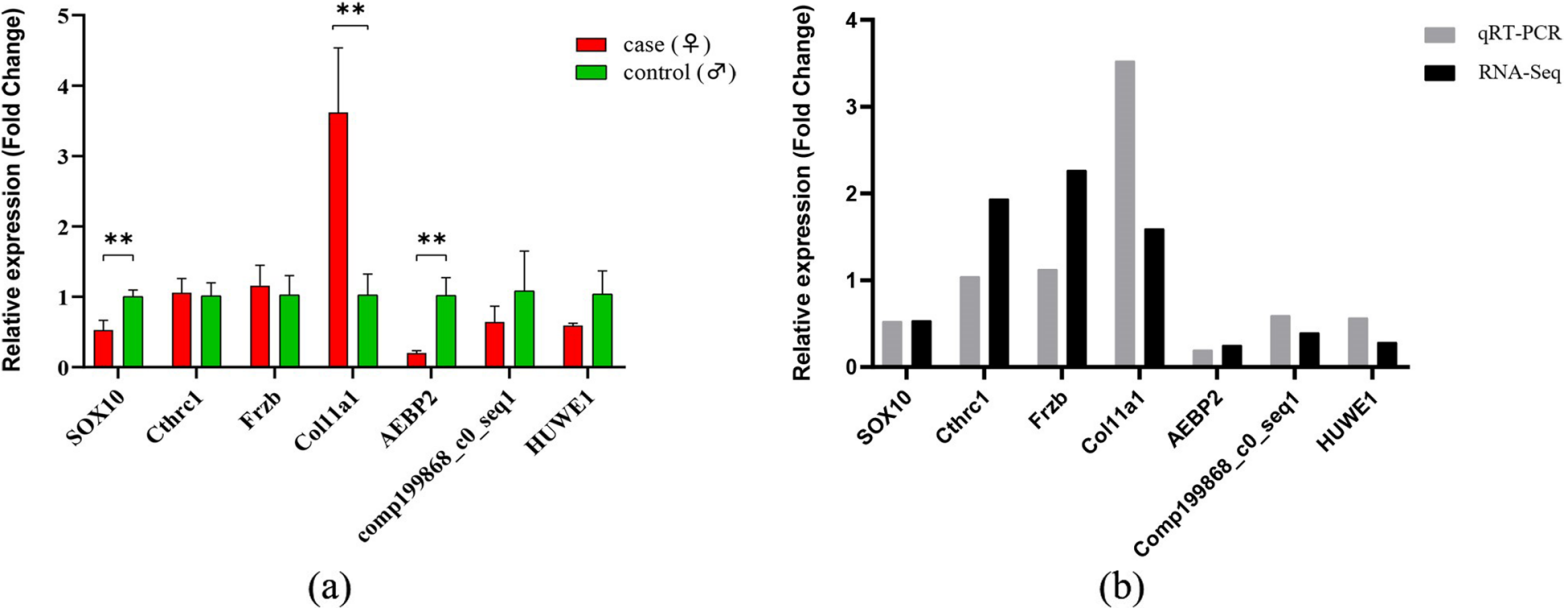
Validation of seven differentially expressed genes in *O. tormota* by qRT-PCR. **a** The expression profiles of DEGs between sexes of *O. tormota*. The horizontal axis represents seven selected DEGs and the error bars represents the mean ± SD of samples (*N* = 3). **b** Comparison of the expression profiles between qRT-PCR and RNA-Seq

To validate the expression profiles of DEGs obtained via Illumina sequencing, seven DEGs were selected for qRT-PCR. The seven DEGs included four auditory-related genes (i.e., *SOX10*, *Cthrc1*, *Frzb* and *Col11a1*) and other three randomly selected DEGs (i.e., *AEBP2*, comp199868_c0_seq1, and *HUWE1*). Generally, the expression pattern of the RNA-Seq results were consistent with the qRT-PCR validation results (Fig. 5), suggesting the accuracy and reliability of the transcriptome data of *O. tormota* in the present study.

## Discussion

In the present study, we compared the gene expression divergence between male and female *O. tormota*, and aimed to identify the related genes involved in the high-frequency hearing. We identified eleven potential high-frequency hearing candidate genes involved in the sound signal pathway and other related regulatory function. In addition, the differential expression patterns of these candidate genes suggested that males showed a higher expression trend than females in both quantity and expression quantity. The highly expressed genes in males were relatively concentrated in neurogenesis, signal transduction, ion transport and energy metabolism, whereas the up-expressed genes in females were mainly related to the growth and development regulation of specific auditory cells. Our results here will provide insights for understanding the genetic changes underlying the sexual difference of ultrasonic hearing in *O. tormota*.

### Sound perception and neurogenesis

Three differentially expressed genes (i.e., *Cthrc1*, *Col11a1* and *Frzb*) were found to be involved in auditory hair cell stereocilium organization and sensory perception of sound. The *Cthrc1* is a Wnt cofactor protein that selectively activates the planar cell polarity (PCP) pathway to regulate the arrangement of ciliary bundles on sensory hair cells of the cochlea [31]. In addition, *p*revious analyses shown that *Frzb* is a Wnt antagonist and could interact with Wnt5a to regulate the otic capsule formation [32]. Furthermore, the gene *Col11a1* (Collagen alpha-1 (XI) chain) have been detected in mouse and other vertebrate mammals cochlear (e.g., tectorial membranes/basilar membranes). Its mutations caused auditory loss and affected the high frequency sensorineural hearing [33, 34]. We found the expression of these three genes were higher in females than that in males. Further studies should be conducted to explore the detailed roles of these genes in the brain during the high-frequency hearing in frogs.

Another DEG belongs to the SRY-related high mobility group box (SOX) family, *SOX10*, plays an important role in neurogenesis [35–37]. Humans with mutations in the *SOX10* gene resulted in sensorineural hearing defects and auditory-pigmentary disorder [36]. Functional enrichment analyses in the present study further suggested the important role of *SOX10* in regulating the formation of neural crest cell and auditory perception related neurons. We also found the expression of *SOX10* in male *O. tormota* was significantly up-regulated compared with females (*P* < 0.01), reflecting the difference of differentiation and regulation of hearing-related neurons between sexes of *O. tormota*.

In addition, the hairy/enhancer of split 5 gene (*Hes5*) is significantly expressed in the auditory sensory epithelial cells of most vertebrates [38, 39] and it is an essential negative regulator of neurogenesis [40, 41]. Evidences shown that *Hes5* can negatively regulate the expression of notch through the Notch signaling pathway and control the normal occurrence of auditory receptor cell [42]. Previous studies have also suggested that loss of *Hes5* in mice can lead to overproduction of hair cells during the embryonic development, which would affect the normal transduction of electrical signals for mechanical energy and the processing of information by the downstream central nervous system [4, 40, 43]. In this study, the significantly higher expression of the *Hes5* gene in male *O. tormota* than that in females were found, and it might indirectly affect the occurrence and movement of auditory receptor cell between different sexes. We speculated that this might regulate the sensitivity of basilar papilla to high-frequency sound signals and these sensory cells exhibited the auditory differences between male and female *O. tormota* during the process of signal transduction to the central auditory nervous system [44].

### Reactive oxygen species (ROS)

Accumulating evidence suggests that the ambient loud noise tend to cause excessive accumulation of reactive oxygen species (ROS) and produce damages to the auditory system, especially the high-frequency auditory sensitive areas in the central nervous system of brain [4, 45]. The antioxidant capacity of the auditory system appears to be important for successful hearing. It has been suggested that the glutathione peroxidase 1 (*Gpx1)* played key role in reducing oxidative damage of cells via regulating cell growth and redox process [46–48]. In the current study, the expression of *Gpx1* in female *O. tormota* was significantly up-regulated compared with males. Although female frogs invested lots of energy in reproduction during the breeding season, they also needed more oxidative capacity to maintain the energy expended for calling (up to 16 kHz) [19]. By contrast, the males’ reproductive activity was more “relaxed” by adopting energy-saving coping strategies to increase the frequency of calls to resist background noise interference and warn competitors [49]. The higher expression of *Gpx1* in females suggested that females improved the ability to repair hearing damage caused by noise interference, whereas males evolved the ultrasound communication capabilities to adapt the ambient noise. More detailed regulation mechanism of the antioxidant capacity between males and females needs to be further studied in the future.

### Ion transport and signal transduction

The transformation of the mechanical signals to ion fluxes and related signal transduction are essential for hearing [50, 51]. Five DEGs (i.e., *PHOX2B*, *KCNK1*, *TECTA*, *TIMM13* and *PAFAH1B1*) were identified and they play key roles in ion transport and electronic signal transduction. The paired-like homeobox 2b (*PHOX2B*) is essential for the differentiation of the auditory efferents neurons and innervating the sensory signals transduction in the hindbrain [52–54]. The significantly higher expression of *PHOX2B* in males might suggest its important role in the sensitivity and coding of high-frequency signals.

There is increasing evidence that the potassium ion channel encoding gene (*KCNQ4*), motor protein (*Prestin*) and other key genes involved in the sound signal transduction (i.e., *TMC1*, *CDH23*, *Pcdh15* and *Otof*) undergone adaptive evolution in echolocation bats, and were strongly associated with the high-frequency hearing [24, 27, 55–57]. However, these genes exhibited no significant expression difference between the sexes of *O. tormota*. Interestingly, another member of the potassium ion channel, *KCNK1*, was up-regulated expressed in males than females. *KCNK1* plays important role in auditory efferent neurons and it has the similar function to *KCNQ4*, which can promote the electrical movement of cells through the change of membrane potential and amplify the auditory sensitivity and frequency selectivity [58, 59]. Functional enrichment analysis further convinced the roles of *KCNK1* in potassium ion transmembrane transport. Thus, the higher expression of *KCNK1* in male *O. tormota* might facilitate the ultrasonic hearing.

Besides, the *TECTA* gene encodes alpha-tectorin (a major non-collagenous component of the tectorial membrane) and plays significant roles in the conductivity of high-frequency sound waves [60, 61]. In addition, mutations in human *TECTA* lead to nonsydromic hearing loss [61]. The significantly higher expression of *TECTA* in males might contribute to the transduction of high-frequency sound signals into electrical signal and benefit the ultrasonic hearing. The differential expression of *TIMM13* and *PAFAH1B1* between the sexes of *O. tormota* should be further investigated.

### Endocrine regulation and energy metabolism

Previous studies have demonstrated that endocrine system and energy metabolism can influence sound signal production and reception via modulating the vertebrate's response and sensitivity to auditory signals [62–65]. There is evidence that neuropeptide hormones (e.g., arginine) and other steroid hormones (e.g., androgens and estrogens) can modulate and induce the advertisement callings or vocalizations in male frogs during the breeding season [64]. A recent work found that female green treefrogs (*Hyla chrysoscelis*) significantly increased the midbrain’s auditory thresholds in response to the frequency of male advertisement callings after injection with the testosterone [66]. In this work, we found that some DEGs were significantly enriched to “Response to steroid hormone” (GO:0048545), “Steroid hormone binding” (GO:1990239), and “Gonadotropin hormone-releasing-hormone activity” (GO:0005183). Among them, most DEGs were significantly up-regulated in males, which might suggested the important role of the hormones in regulation of the sensitivity to high-frequency hearing.

In addition, the metabolism-related biological functions or signaling pathways were significantly enriched in large quantities based on the functional enrichment analysis. Generally, most female frogs put more efforts into spawning or performing special energy-consuming reproductive behaviors, and they were regarded as relatively “silent” during reproduction [62, 67]. The physiological activities (e.g., energy production and metabolism) might contribute to the high-frequency hearing difference between the sexes of *O. tormota* via affecting the brain-processing and response to acoustic signals. In the present study, most of the DEGs were up-regulated in male *O. tormota* compared with females, and these DEGs are involved in Glycolysis/Gluconeogenesis (ko00010) processes, biosynthesis and metabolism of amino acids (ko01230), and other energy consuming processes (e.g., Oxidative phosphorylation: ko00190). Evidences shown that male *O. tormota* can communicate with intraspecific species through high-frequency acoustic signals, which required greater energy expenditure to accomplish [19, 22]. Sound playbacks and electrophysiological experiments have found that female frogs preferred males with a small body size and a louder calls to complete mating [20, 68]. Thus, males *O. tormota* might have evolved higher energy metabolic systems to enhance their own calls frequency and mate attraction. Further study should be focused on the endocrine and energy metabolism related genes, and their relationship to acoustic communication.

## Conclusions

In general, these results demonstrated that ultrasonic hearing is a complex network involving multiple metabolic and physiological pathways. We used comparative transcriptome analysis to characterize a number of novel candidate genes associated with high-frequency hearing in *O. tormota*. Ultrasonic hearing is highly correlated with neurogenesis, ion transport, signal transduction, endocrine regulation and energy metabolism. Our results here will provide insights for understanding the genetic changes underlying the sexual difference of ultrasonic hearing in *O. tormota*.

## Methods

### Sampling, RNA extraction and sequencing

Samples of *O. tormota* were collected from the Huangshan mountain, Anhui province, China in April 2016. A total of 12 adult individuals (eight males and four females) were collected and they were randomly divided into two male (named M1 and M2) and two female (named F1 and F2) groups. Each male and female group consisted of four and two individuals, respectively. These frogs were euthanized by tricaine methanesulfonate (MS-222) and sacrificed to collect brain tissues. All brain tissues were frozen in liquid nitrogen for 3 h and then stored at -80℃ for further use. All animal sampling and use protocols were conducted in accordance with all the ethical guidelines and legal requirements in China, and were approved by the Institutional Care and Ethics Committee of Henan Normal University.

Total RNA of each sample was extracted separately from the brain tissue using TRIzol Reagent (Invitrogen, Carlsbad, CA, USA) according to the manufacturer’s instructions. Equal amounts of total RNA from each individual were pooled together for each group (i.e., F1, F2, M1 and M2) and then used for library construction and sequencing. The RNA degradation and contamination was determined by 1% agarose gel electrophoresis. The RNA purity and integrity were assessed using the NanoDrop 2000 spectrophotometer (Thermo Scientific, USA) and the Agilent 2100 Bioanalyzer (Agilent Technologies, Santa Clara, CA, USA), respectively. After digested by DNase and purified with poly-T oligo-attached magnetic beads, the mRNAs were fragmented. First-strand complementary DNA (cDNA) was synthesized using random hexamer primers and M-MuLV Reverse Transcriptase (RNase H). The second-stand cDNA was subsequently synthesized with DNA Polymerase Ι and RNase H. Subsequently, double-stranded cDNA was further subjected to end-repair and ligation with adapters. The final cDNA library was constructed using TruSeq Stranded mRNA LT Sample Prep Kit (Illumina, San Diego, CA, USA) according to the manufacturer’s instructions. After testing the quality of the libraries, they were sequenced on the Illumina HiSeq^TM^2500 platform and paired-end reads were generated at OE Biotech Co., Ltd., Shanghai, China.

### De novo assembly and functional annotation

Before assembly, the reads quality was evaluated using FASTQC software (http://www.bioinformatics.babraham.ac.uk/projects/fastqc/). Reads containing adaptors, reads containing poly-N and low quality reads were removed by NGS QC Toolkit v2.3.3 (http://59.163.192.90:8080/ngsqctoolkit/) [69]. Quality parameters of clean data including Q30 and GC-content were obtained at the same time. All the subsequent analyses were carried out using high quality clean reads. Due to the lack of reference genome sequences for *O. tormota*, the total high-quality clean reads from all samples were assembled de novo using Trinity (https://github.com/trinityrnaseq/trinityrnaseq/wiki) with default parameters. All the redundancy sequences were removed using TGICL software and further produced the longest unigenes [70]. All the unigenes from the four groups were combined and used as the reference sequences for subsequent analyses.

The assembled reference sequences were aligned to NCBI non-redundant protein sequence database (NR), the manually annotated and reviewed protein sequence database (Swiss-Prot; http://www.ebi.ac.uk/swissprot/) and the eukaryotic Ortholog Groups (KOG) (http://www.ncbi.nlm.nih.gov/COG) using BLASTx with a threshold E-value of 10^–5^. For each unigene, the best BLASTx hit from the NR database was submitted to the Blast2GO [71], and Gene Ontology (GO) terms were obtained based on annotations between GO terms and gene names. The Kyoto Encyclopedia of Genes and Genomes (KEGG) Automatic Annotation Server (KAAS) was used for the KEGG pathway annotation and assignments [72].

### Differentially expressed genes (DEGs) and pathway enrichment analysis

The clean reads of each sample were aligned to the assembly using Bowtie2 [73], and the resulting alignments were used to estimate the unigene expression abundance [74]. We used false discovery rate (FDR) [75] and fragments of exon per kb per million fragments (FPKM) value [76] to estimate the *p*-value threshold and gene expression levels, respectively. The DEGseq R package was applied to filter the differentially expressed genes (DEGs) with a fold change greater than 2 (|log2Fold Change|> 1) and *P*-value (FDR) less than 0.05 [77]. Hierarchical cluster analysis of DEGs was further performed to show the gene expression pattern in different groups.

To elucidate the biological implications of DEGs and reveal whether those DEGs are involved in special auditory adaptation, GO and KEGG pathway enrichment analyses of the DEGs were performed. Based on the Wallenius’ non-central hypergeometric distribution, GO enrichment analysis of the DEGs was conducted. KEGG pathway enrichment of the DEGs was tested using KOBAS software (http://kobas/cbi.pku.edu.cn/home.do). Fisher’s exact test was used to identify the significant GO categories and KEGG enrichment pathway, and FDR was used to correct the *p*-values [78]. The threshold of significance was defined by *p*-value < 0.05 and FDR < 0.05.

### Quantitative real-time PCR (qRT-PCR) validation

To validate the accuracy of our transcriptome expression profiles, quantitative real-time PCR (qRT-PCR) was conducted. Seven of the DEGs revealed in this study were randomly selected for the validation by qRT-PCR, and the primers were listed in Additional file 1: Table S1. *GADPH* (glyceraldehydes-3-phosphate dehydrogenase) was used as the internal control. The qRT-PCR was performed using TB Green® Premix EX Taq™ II (TaKaRa, Beijing, China) according to the manufacture’s protocol with the LightCycler96 Real Time PCR system (Roche, Switzerland). Each sample was detected in triplicate. The relative gene expression levels of candidate genes to reference gene were analyzed using the 2^−△△Ct^ method and presented as fold changes for the calibrator [79], and then the Unpaired Student’s t-test was used to analyzed significances for qRT-PCR data.

## Supplementary Information

**Additional file 1: Table S1. **Primers used for qRT-PCR amplification.

**Additional file 2: Table S2. **De Novo assembly statistics for the brain transcriptome in *O. tormota*.

**Additional file 3: Figure S1**. Length distribution statistics of the assembly All-unigene. Horizontal axis representsthe All-unigene length interval; Vertical axis indicates the number of All-unigene within the length interval range.

**Additional file 4: Table S3.** Statistical result of clean reads mapped against the assembled unigenes.

**Additional file 5: Figure S2.** Species distribution based on the best hit of NR blast result. Different species are represented in different colors, and the size of the pie region represents the proportion of unigenes which were annotated to different species.

**Additional file 6: Figure S3.** KOG classification of the Unigenes in *O. tormota*. Different KOG function classes are shown in different letter and colors, the number of genes are shown along the vertical axis.

**Additional file 7: Figure S4.** Heat map and hierarchical clustering of differential expressed genes in four groups. The red represents up-regulated unigenes, and down-regulated unigenes are represented in green.

**Additional file 8: Table S4.** Number of DEGs between different groups in *O. tormota*.

**Additional file 9: Table S5.** 4605 differentially expressed genes in the brain between female and male *O. tormota*.

**Additional file 10: Table S6.** Statistical summary of KEGG pathway enrichment analysis at different hierarchies.

**Additional file 11: Table S7.** Summary of significantly differentially expressed genes related to auditory identified in the brain transcriptome.

## Data Availability

The data sets of sequencing reads have been deposited in the National Center for Biotechnology Information database (NCBI) and can be retrieved under the GEO accession number GSM5691958, GSM5691959, GSM5691960, and GSM5691961.

## Abbreviations

BP: Biological Process
CC: Cellular Component
DEGs: Differentially Expressed Genes
FPKM: Fragments of exon per kb per million fragments
FDR: False Discovery Rate
GO: Gene Ontology
KAAS: KEGG Automatic Annotation Server
KEGG: Kyoto Encyclopedia of Genes and Genomes
KOG: Eukaryotic Ortholog Groups
MF: Molecular Function
NCBI: National Center for Biotechnology Information
NR: Non-redundant protein sequence database
PCP: Planar Cell Polarity pathway
Ptor: Principal nucleus of the torus semicircularis
qRT-PCR: Quantitative real-time Polymerase Chain Reaction
ROS: Reactive Oxygen Species
SON: Superior Olivary Nucleus
