# Supplementary figures and images for: Comparative transcriptome analysis provides insights into the molecular mechanisms of high-frequency hearing differences between the sexes of Odorrana tormota
Source: BMC Genomics. 2022 Apr 12;23:296. doi: 10.1186/s12864-022-08536-2 (PMC9004125; doi:10.1186/s12864-022-08536-2)

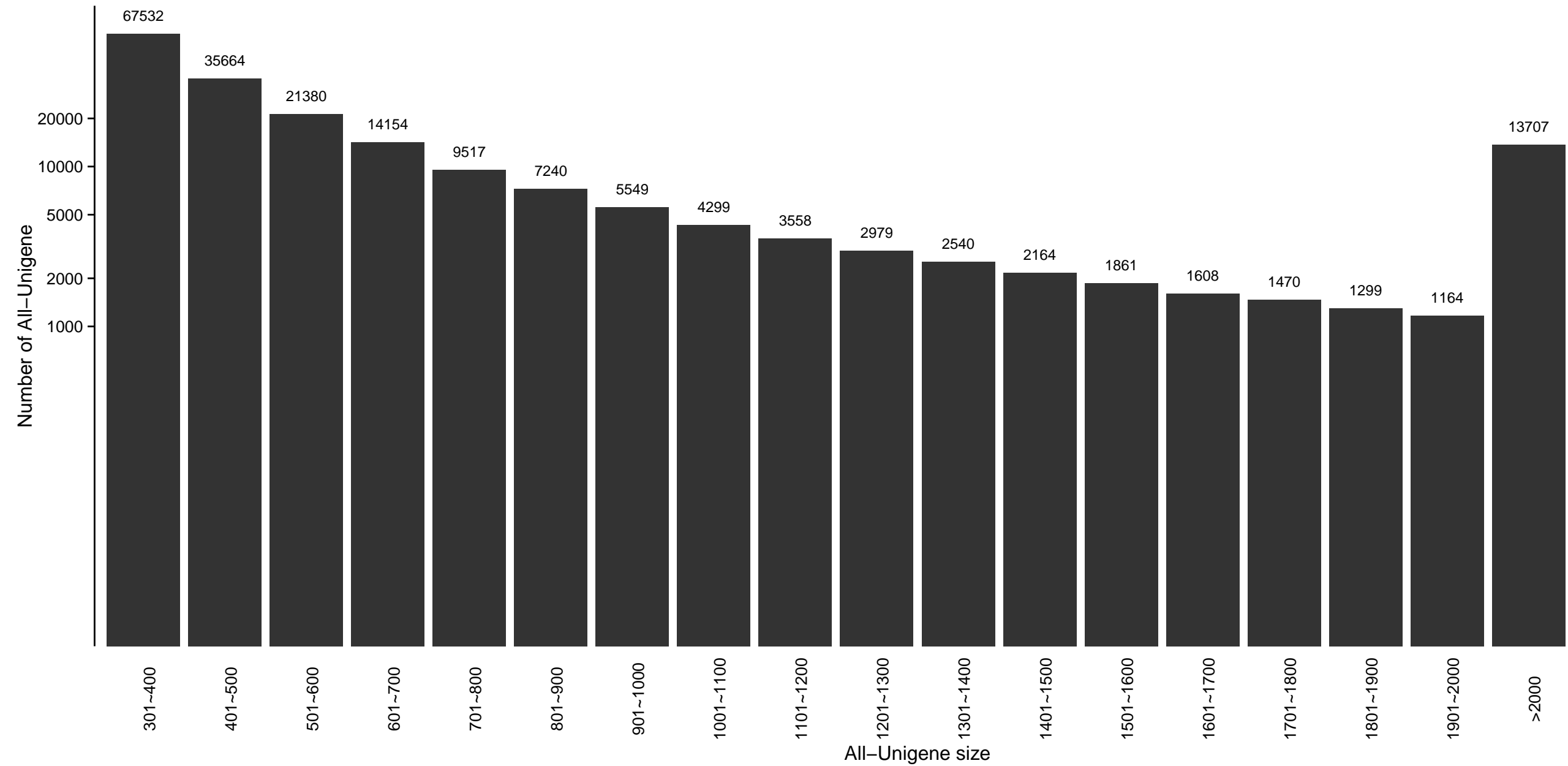

Supplement: Supplementary file 3 — Additional file 3: Figure S1. Length distribution statistics of the assembly All-unigene. Horizontal axis representsthe All-unigene length interval; Vertical axis indicates the number of All-unigene within the length interval range. [file 12864_2022_8536_MOESM3_ESM.pdf]

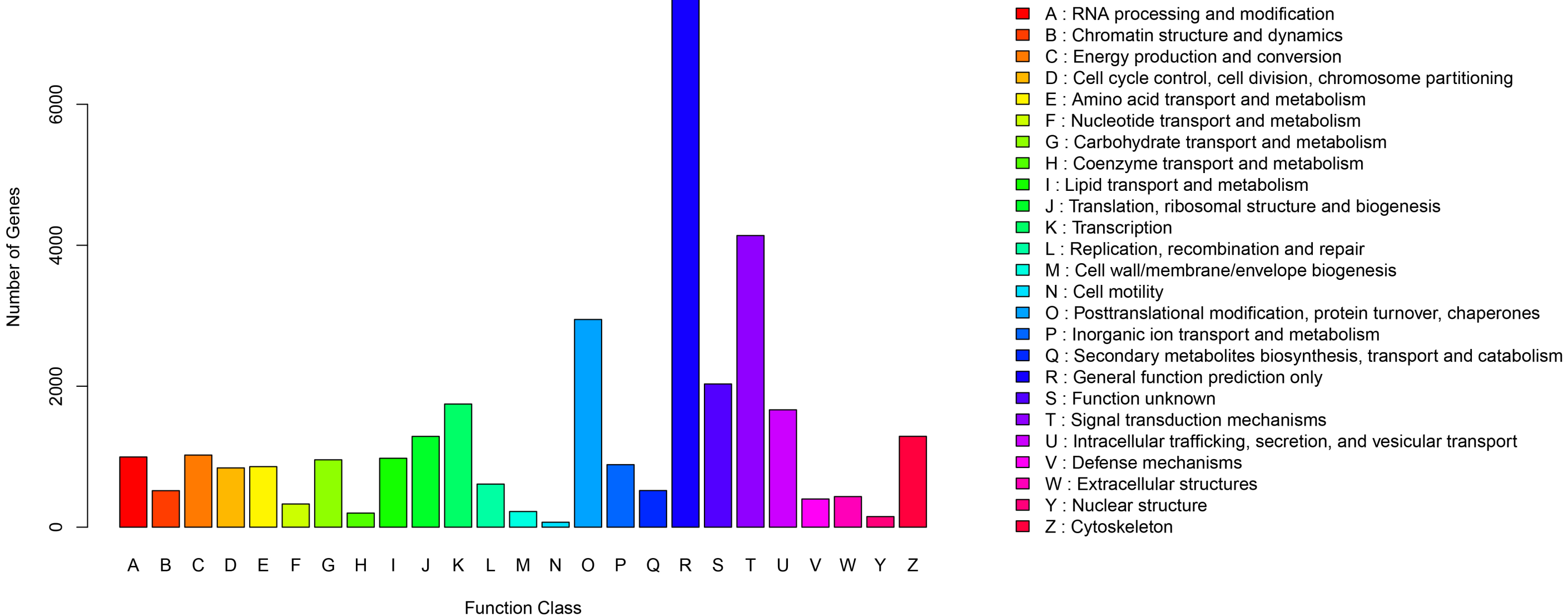

Supplement: Supplementary file 6 — Additional file 6: Figure S3. KOG classification of the Unigenes in O. tormota. Different KOG function classes are shown in different letter and colors, the number of genes are shown along the vertical axis. [file 12864_2022_8536_MOESM6_ESM.pdf]

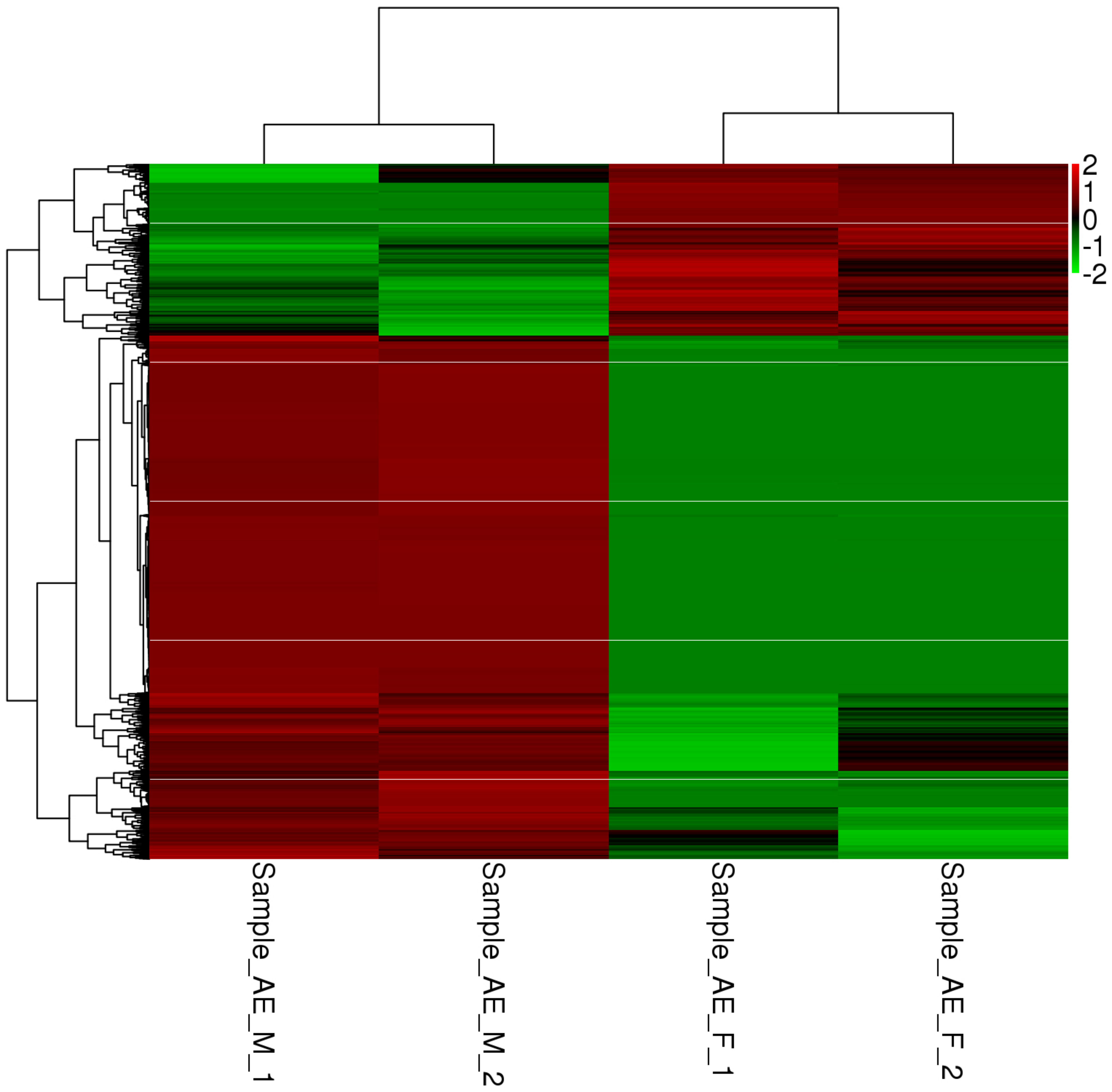

Supplement: Supplementary file 7 — Additional file 7: Figure S4. Heat map and hierarchical clustering of differential expressed genes in four groups. The red represents up-regulated unigenes, and down-regulated unigenes are represented in green. [file 12864_2022_8536_MOESM7_ESM.pdf]
