# Supplementary material for: Comparative transcriptome analysis provides insights into the molecular mechanisms of high-frequency hearing differences between the sexes of Odorrana tormota
Source: BMC Genomics. 2022 Apr 12;23:296. doi: 10.1186/s12864-022-08536-2 (PMC9004125; doi:10.1186/s12864-022-08536-2)
Supplement: Supplementary file 5 — Additional file 5: Figure S2. Species distribution based on the best hit of NR blast result. Different species are represented in different colors, and the size of the pie region represents the proportion of unigenes which were annotated to different species. [file 12864_2022_8536_MOESM5_ESM.pdf]

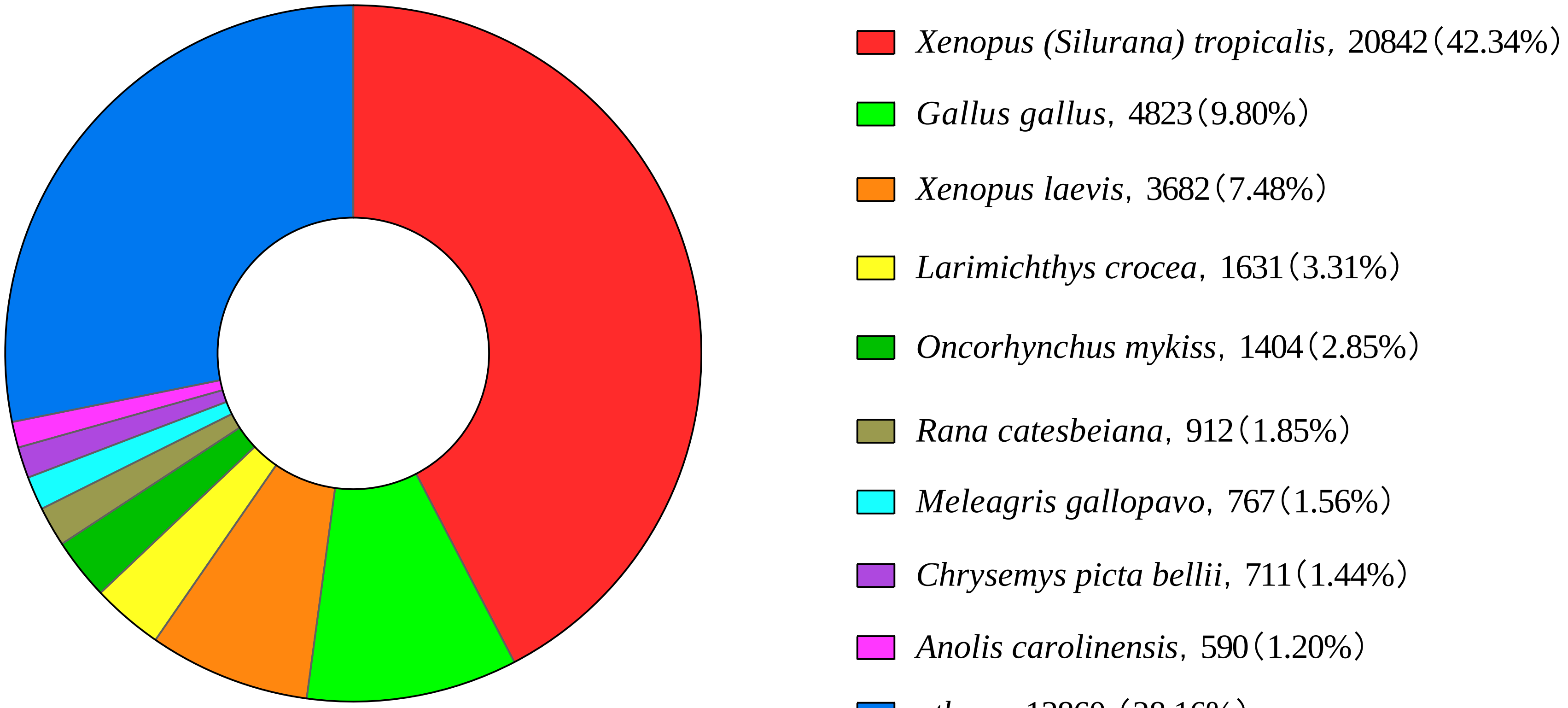

Xenopus (Silurana) tropicalis, 20842（42.34%）
Gallus gallus, 4823（9.80%）
Xenopus laevis, 3682（7.48%）
Larimichthys crocea, 1631（3.31%）
Oncorhynchus mykiss, 1404（2.85%）
Rana catesbeiana, 912（1.85%）
Meleagris gallopavo, 767（1.56%）
Chrysemys picta bellii, 711（1.44%）
Anolis carolinensis, 590（1.20%）
others, 13860（28.16%）
